# Effects of substrates on N_2_O emissions in an anaerobic ammonium oxidation (anammox) reactor

**DOI:** 10.1186/s40064-016-2392-1

**Published:** 2016-06-16

**Authors:** Yue Jin, Dunqiu Wang, Wenjie Zhang

**Affiliations:** Guangxi Key Laboratory of New Energy and Building Energy Saving, College of Civil Engineering and Architecture, Guilin University of Technology, 12, Jiangan Road, Guilin, 541004 China; Guangxi Key Laboratory of Environmental Pollution Control Theory and Technology, Guilin University of Technology, Guilin, 541004 China; Guangxi Collaborative Innovation Center for Water Pollution Control and Water Safety in Karst Area, Guilin University of Technology, Guilin, 541004 China

**Keywords:** Substrate concentrations, NH_4_-N, Anammox, N_2_O

## Abstract

N_2_O emission in the anaerobic ammonium oxidation (anammox) process is of growing concern. In this study, effects of substrate concentrations on N_2_O emissions were investigated in an anammox reactor. Extremely high N_2_O emissions of 1.67 % were led by high NH_4_-N concentrations. Results showed that N_2_O emissions have a positive correlation with NH_4_-N concentrations in the anammox reactor. Reducing NH_4_-N concentrations by recycling pump resulted in decreasing N_2_O emissions. In addition, further studies were performed to identify a key biological process that is contributed to N_2_O emissions from the anammox reactor. Based on the results obtained, *Nitrosomonas*, which can oxidize ammonia to nitrite, was deemed as the main sources of N_2_O emissions.

## Background

Nitrogen removal (NR) is an important component of wastewater treatment. Biological nitrogen removal (BNR) is often preferred to other non-biologic processes due to its high efficiency and energy conservation characteristics. Even so, the traditional BNR process has several disadvantages, such as excessive oxygen being consumed during the nitration period and the requirement of additional organic carbon for denitrification. In 1995, the biological anaerobic ammonium oxidation (anammox) reaction was first reported in an up-flow reactor (Mulder et al. 1995). The anammox process operates under anaerobic conditions where nitrite is used as an electron acceptor by anammox bacteria for oxidation of ammonia to nitrogen gas (N_2_) (Kuenen 2008). By using this new technology, only 50 % of the source ammonium needs to be oxidized to nitrite. This means that the oxygen requirement is reduced to about 75 % of the traditional BNR process. Anammox bacteria are autotrophic microorganisms, therefore, additional carbon input is also eliminated. The anammox process has demonstrated potential over the traditional BNR process, thus considerable research has been carried out from bench-scale to pilot-scale as the technology has proceeded to full-scale applications (Kartal et al. 2010).

N_2_O is a potent greenhouse gas, whose warming effect is 200–300 times that of CO_2_ and 4–12 times greater than CH_4_. Many studies have shown that standard sewage denitrification processes are a critical source of atmospheric N_2_O (Kampschreur et al. 2009a; Wunderlin et al. 2013; Shaw and Koh 2012). In addition, research has generally shown that N_2_ is the end product of the anammox process (Jetten et al. 2005); however, high N_2_O emission from Anammox processes have also been reported (Kampschreur et al. 2009b). Thus, there is an urgent need to investigate the production of N_2_O in the anammox process and develop methods of controlling and decreasing the greenhouse emissions from the anammox process.

In this study, an anammox reactor was used to study the effects of substrate concentrations on the emission of N_2_O in an anammox process. The relationship between substrate concentrations and N_2_O emissions was studied by changing the influent NH_4_-N concentration. Furthermore, genetic analysis using the 16S rRNA gene was employed to characterize the microbial population of the anammox granules.

## Results and discussion

### Reactor performance

**Fig. 1:**
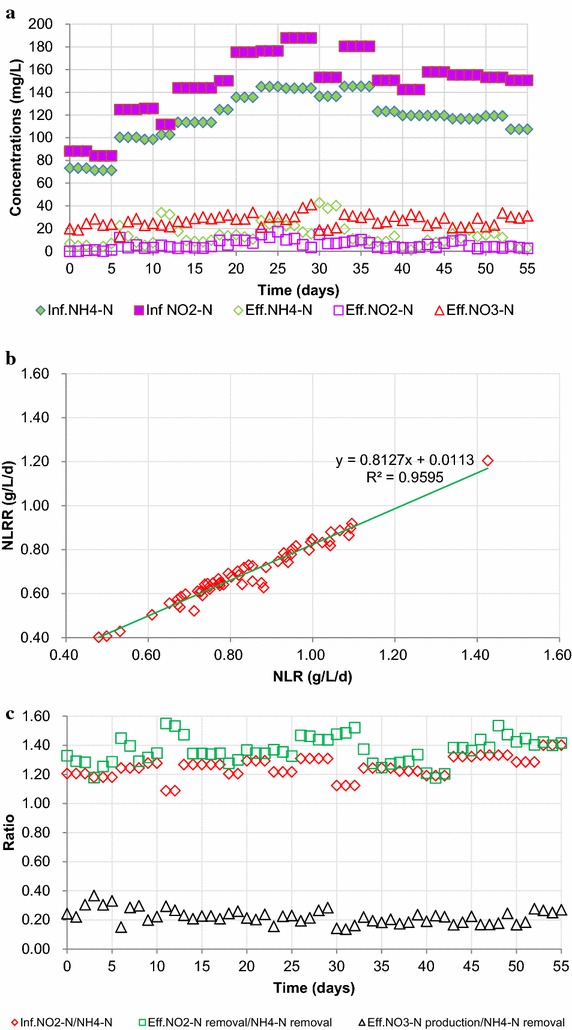
Reactor performance during the study. **a** Changes in nitrogen concentrations; **b** changes in NLR and NLRR; **c** ratios of inf. NO_2_-N/NH_4_-N, eff. NO_2_-N removal/NH_4_-N removal, and eff. NO_3_-N production/NH_4_-N removal. *Inf.* influent, *Eff.* effluent

The removal performance of ammonia and nitrite is shown in Fig. 1a, b. Whenever the effluent NO_2_**-**N concentration fell below 10 mg L^−1^, the nitrogen loading rate (NLR) was increased by adjusting the influent nitrogen concentration while maintaining a constant HRT of 8 h. During start-up period, with the influent NH_4_**-**N and NO_2_**-**N concentrations set at 73.2 and 88.3 mg L^−1^, respectively, effluent NH_4_**-**N and NO_2_**-**N concentrations below 7 and 2 mg L^−1^ were obtained, with the TN removal rate >80 %. Subsequently, at a constant HRT, the influent NH_4_**-**N and NO_2_**-**N concentrations were further increased to 100.4 and 124.8 mg L^−1^, respectively, and effluent NH_4_**-**N and NO_2_**-**N concentrations initially were a little higher, but both soon decreased to below 8 mg L^−1^ over a 3-day period. These results indicated that the seed anammox sludge could adapt quickly to changes in NLR. On day 24, the influent NH_4_**-**N and NO_2_**-**N concentrations were increased to 145.0 and 176.4 mg L^−1^, respectively, which were the highest levels used in this study. Under these conditions, the effluent NH_4_**-**N and NO_2_**-**N concentrations were 27.0 and 14.8 mg L^−1^, respectively. Accordingly, influent NH_4_**-**N and NO_2_**-**N concentrations were decreased to 120 and 150 mg L^−1^, respectively, and effluent NH_4_**-**N and NO_2_**-**N concentrations were then maintained below 18.6 and 9.9 mg L^−1^. Overall, the reactor could operate with a stable nitrogen removal rate of over 81 %.

Figure 1c shows the ratios of influent NO_2_**-**N/NH_4_**-**N, effluent NO_2_-N removal/NH_4_-N removal, and effluent NO_3_-N production/NH_4_-N removal. At the start-up period, influent NO_2_**-**N/NH_4_**-**N was set 1.2. Accordingly, effluent NO_2_-N removal/NH_4_-N removal, and Effluent NO_3_-N production/NH_4_-N removal were 1.3 and 0.3, respectively, which were close to values reported by others (Strous et al. 1998). In order to investigate the effect of influent NH_4_-N on N_2_O emission, on day 12 influent NO_2_**-**N/NH_4_**-**N was changed to 1.09. As a result, effluent NO_2_-N removal/NH_4_-N removal increased to 1.55. Conversely, effluent NO_3_-N production/NH_4_-N removal decreased to 0.2. The same results were once more affirmed on day 31. Denitrification was considered to be the main reason for the additional NO_2_-N or NO_3_-N removal.

### N_2_O emission

**Fig. 2:**
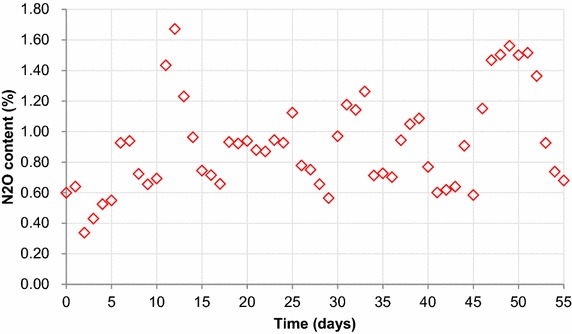
N_2_O emission during the study

N_2_O emissions over the course of the study are shown in Fig. 2. The conversion ratio of N_2_O was calculated from the removed nitrogen. On the first day, about 0.6–0.64 % N_2_O content was detected in the emission gas. On day 2, the influent pipe became blocked, thus only 0.34 % N_2_O was detected in the emission gas. However, this value increased to 0.54 % over the following 3 days and by day 6 the N_2_O concentration had reached 0.93 %, accompanied with a high effluent NH_4_-N residual. On days 11–13 and 30–32, the effluent NH_4_-N remained at 32–34 and 37–42 mg L^−1^, respectively. Under these conditions, the N_2_O emissions were found to be significantly higher than the values associated with low effluent NH_4_-N concentrations. Over the course of the study, N_2_O levels were determined to be 0.6–1.0 % in the off-gas.

### Effects of influent NH_4_-N, NO_2_-N, NO_3_-N and nitrogen removal rate on N_2_O emission

**Fig. 3:**
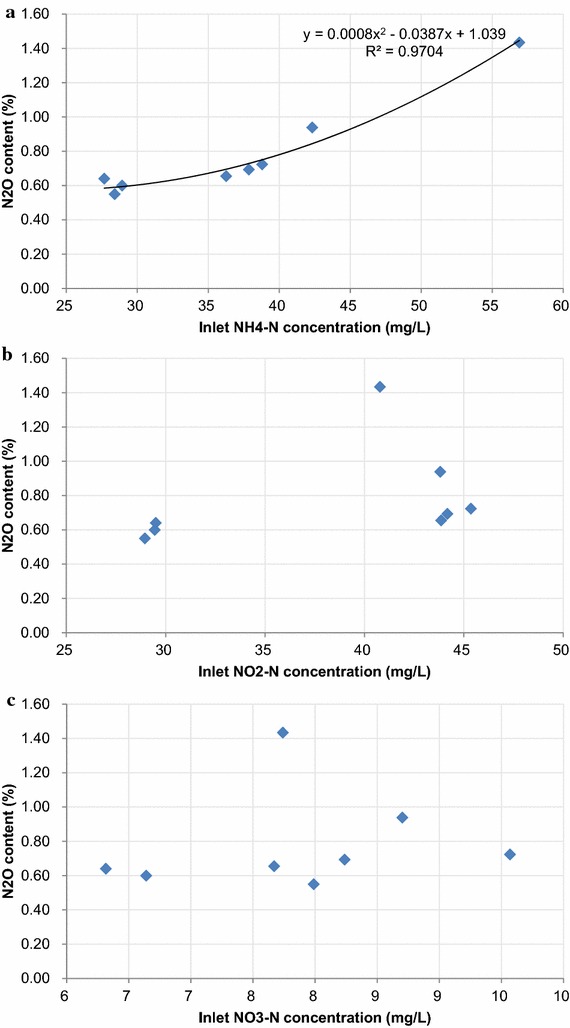
Effect of substrate on N_2_O emission. **a** Relation of N_2_O emission and inlet NH_4_-N concentration; **b** relation of N_2_O emission and inlet NO_2_-N concentration; **c** relation of N_2_O emission and inlet NO_3_-N concentration

Effects of inlet NH_4_-N, NO_2_-N, NO_3_-N and nitrogen removal rate on N_2_O production are shown in Fig. 3. The EGSB reactor used in this study was operated with a high recycle rate. Thus, influent NH_4_-N, NO_2_-N and NO_3_-N were calculated by using the following equation.

1 $$x = \frac{a + n \times b}{n + 1}$$

where *x* is the concentration of inlet NH_4_-N, NO_2_-N, NO_3_-N, *a* is the influent concentration of NH_4_-N, NO_2_-N, NO_3_-N, *n* is the ratio of recycle rate to influent flow rate, *b* is the effluent concentration of NH_4_-N, NO_2_-N, NO_3_-N. According to the equation, inlet NH_4_-N, NO_2_-N and NO_3_-N were determined by two factors: changing influent concentrations or different recycle rate. In order to observe the effects of inlet NH_4_-N, NO_2_-N and NO_3_-N, only the recycle rate was changed while the nitrogen loading rate was set with the same value 0.5 kg m^−3^ day^−1^ (Fig. 3a–c). Also, the effects of nitrogen removal rate were evaluated with the same influent nitrogen concentrations.

As shown in Fig. 3a, average N_2_O content was 0.6 % with an inlet NH_4_-N concentration of 27–28 mg L^−1^. Increasing the inlet NH_4_-N concentration from 36 to 57 mg L^−1^, N_2_O increased from 0.65 to 1.4 %. Inlet NH_4_-N concentration and N_2_O emission were simulated according to the current data by the following equation with P < 0.03.

2 $$y = 0.0008x^{2} - 0.0387x + 1.039$$

where *y* is the N_2_O emission, *x* is the inlet NH_4_-N concentration. In a word, increasing inlet NH_4_-N concentration tended to yield a higher N_2_O concentration.

The influences of inlet NO_2_-N and NO_3_-N concentrations were also investigated during the study, though no obvious relationship was found with N_2_O emissions (Fig. 3b, c).

### Bacteria community analysis

**Fig. 4:**
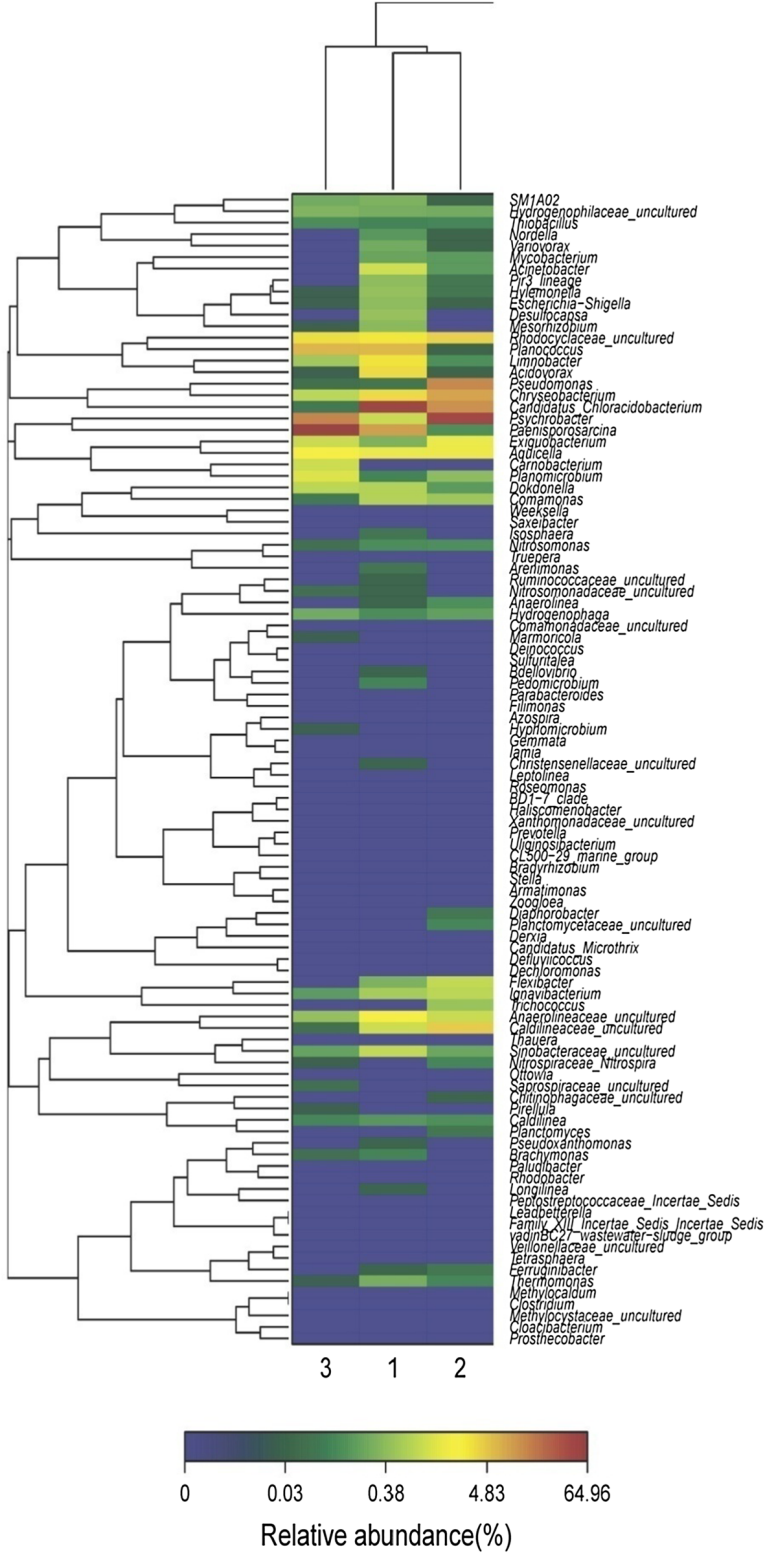
Hierarchical cluster analysis of 1, 2 and 3 bacterial communities. 1, N_2_O emission 0.6 %, nitrogen removal rate 0.4 kg-N m^-3^ day^−1^; 2, day 36, N_2_O emission 0.7 %, nitrogen removal rate 0.73 kg-N m^−3^ day^−1^; 3 day 50, N_2_O emission 0.18 %, nitrogen removal rate 3 kg-N m^-3^ day^−1^. The *y-axis* is the clustering of the 100 most abundant OTUs (3 % distance) in reads. The OTUs were ordered by genus. Sample communities were clustered based on complete linkage method. The *color intensity of scale* indicates relative abundance of each OUT read. Relative abundance was defined as the number of sequences affiliated with that OTU divided by the total number of sequences per sample

**Fig. 5:**
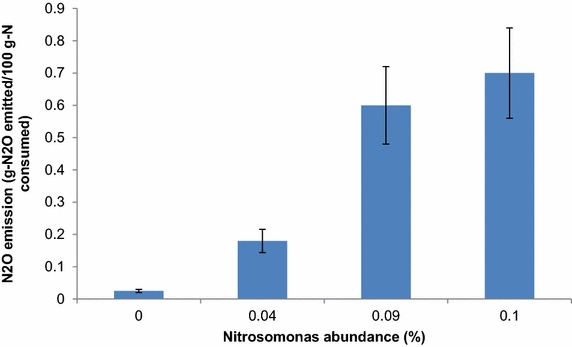
Relationship between *Nitrosomonas* abundance and N_2_O emission

Hierarchical cluster analysis was used to identify the differences of three bacterial community structures (Fig. 4). The three samples were sampled from the same reactor, showing obvious similarity of community structure. *Nitrosomonas*, which oxidizes ammonia to nitrite, was detected in all the three samples. In the anammox reactor, it is difficult to keep dissolved oxygen at zero. Thus, the anammox reactor provides the conditions for the growth of *Nitrosomonas*. However, *Nitrosomonas* is known to produce N_2_O under low oxygen conditions (7). This was supported by the relationship between *Nitrosomonas* abundance and N_2_O emission (Fig. 5).

In this study, N_2_O emissions were found to be higher than the reported values. Okabe et al. reported that a N_2_O emission of only 0.05–0.23 % was detected with a nitrogen removal rate of 7.5–15 kg N m^−3^ day^−1^. However, the highest N_2_O concentration of 1.67 % was quantified in this study, which is compared with other the results in Table 1. From Table 1, increasing nitrogen loading rates showed positive effect on decreasing N_2_O concentrations. Longfei et al. (2015) also reported that the increase of nitrogen loading rate could reduce N_2_O emission and they found it is more seasonable if compare the value of N_2_O production per gram N removal (N_2_O emission/nitrogen removal rate). Although higher nitrogen removal rate helps to reduce the footprint of the anammox system, it was difficult to maintain the stable running under high nitrogen removal rate due to floatation of anammox granules and pipe clogging. On the other hand, Kampschreur et al. also found high N_2_O concentrations with 0.6 % in one full-scale anammox reactor. Thus, increasing NLR is effective in reducing N_2_O emission, but N_2_O emissions are inevitable in an anammox reactor. Reducing N_2_O emission is still a concern for anammox applications.

**Table 1:** Comparison of N_2_O emission for different nitrogen loading rate in wastewater treatment

Reactor type	Reactor volume (L)	Removal rate (kg m^−3^ day^−1^)	N_2_O emission (%)	References
Granules-based	70,000	7.14	0.6	Kampschreur et al.
Granules-based	0.15	7.5–15	0.05–0.23	Okabe et al.
GAC-Granules-based	10	0.8	0.6–1.5	Present work

NO_2_-N and NO_3_-N are the substrates for denitrifiers. It is supposed that N_2_O is produced as an intermediate of incomplete heterotrophic denitrification due to low COD/N ratio (Okabe et al. 2011). However, no relationship was found between NO_2_-N, NO_3_-N and N_2_O emission in this study. Thus, it is difficult to explain the increasing N_2_O emission during this study.

Okabe et al. indicated that denitrification by putative heterotrophic denitrifiers present in the inner part of the granule was considered the most probable cause of N_2_O emission from the anammox reactor. In this study, inlet NH_4_-N showed clear relation to N_2_O emission (Fig. 3). Also, *Nitrosomonas* abundance increased with N_2_O emission (Fig. 5). As shown in Fig. 5, only 0.025 g-N_2_O emitted/100 g-N consumed was observed without *Nitrosomonas*. And the denitrifires were presumed to contribute the above N_2_O emission. After that, *Nitrosomonas* abundance increased with N_2_O emission. At last, 0.7 g-N_2_O emitted/100 g-N consumed was observed, which was almost 30 times of the value without *Nitrosomonas*. The results got in this study showed that *Nitrosomonas* was the main cause of N_2_O emission. *Nitrosomonas* competed with anammox bacteria for NH_4_-N. Because anammox bacteria could not oxidize NH_4_-N without NO_2_-N, therefore, supplying enough NH_4_-N is favorable for *Nitrosomonas*. While oxygen was always insufficient for NH_4_-N oxidation in one anammox reactor, thus, N_2_O produced due to NH_2_OH oxidation (Wunderlin et al. 2013). The results of this study is partly consistent with the literature showing that NH_2_OH oxidation by AOB was considered the most probable cause of N_2_O production (0.6 % of the nitrogen load) in a full-scale Anammox reactor treating sludge reject water (Kampschreur et al. 2009b). Beyond that, this study could not exclude the possibility of N_2_O emission by denitrifiers. Further study was needed to quantify N_2_O emission contributed by denitrifiers and *Nitrosomonas* using real wastewater.

## Conclusions

One anammox reactor was used to investigate the effect of substrate concentrations on N_2_O emissions. The monitoring N_2_O concentrations were determined as 0.6–1.0 % in the emission gas during this study. Increasing inlet NH_4_-N concentration from 36 to 57 mg L^−1^, N_2_O increased from 0.65 to 1.4 %. Reduced inlet NH_4_-N concentrations induced N_2_O emission. The results got in this study suggested that in addition to denitrifiers, *Nitrosomonas* was also a significant cause of N_2_O emissions.

## Methods

### Anammox reactor and substrate

**Fig. 6:**
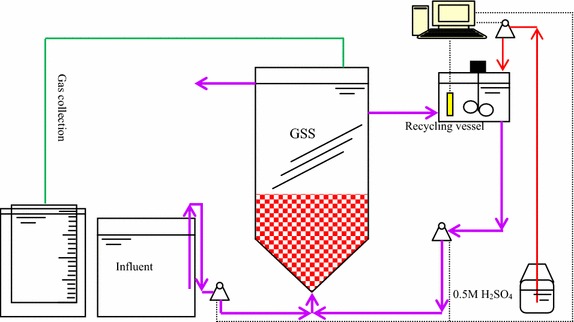
Schematic view of the Anammox reactor system. *GSS* gas solid separator

The reactor had an inner diameter of 14 cm with a total liquid volume of 10 L including a reaction zone of 8 L and a settling zone of 2 L. The reactor was made of acrylic resin and had a water jacket for temperature control. Sampling ports were located at heights of 3, 17, 20 and 25 cm above the reactor bottom. Part of the effluent was collected in a 5-L container (with mixer and heater) for use as recycle (Fig. 6). The pH was adjusted by an online pH controller (TPH/T-10, Tengine, China) using 0.5 mol L^−1^ H_2_SO_4_ (Yue et al. 2015). The reactor was enclosed in a black-vinyl sheet to prevent growth of photosynthetic bacteria and algae.

The reactor was operated in up-flow mode, with influent introduced at the bottom using a peristaltic pump (BT100-2J, LongerPump, China). A recirculation pump (BT600-2J, LongerPump, China) was used to dilute the influent (Fig. 6) with the treated wastewater in the 5-L recycle container.

The anammox seed sludge used in the reactor was taken from a pilot-scale anammox reactor (unpublished). The seed sludge was granule activated carbon (GAC)-based granules with settling velocity over 150 m h^−1^ (Wenjie et al. 2015). The initial seeding concentration (mass of mixed liquor suspended solids (MLSS) per liter) was set at 4 g MLSS L^−1^.

The reactor was fed with synthetic wastewater with a nitrite to ammonium molar ratio of 1.0–1.2. The detailed composition of the influent is shown in Table 2. The influent storage tank was flushed with nitrogen gas to maintain DO under 0.5 mg L^−1^, and Na_2_SO_3_ was added to a concentration of 40 mg L^−1^ (shown to be harmless to Anammox bacteria, Wenjie et al. 2014) to keep the DO level close to zero.

**Table 2:** Composition of synthetic wastewater

Composition	Concentration (mg L^−1^)
(NH_4_)_2_SO_4_, NaNO_2_ (as mg N L^−1^)	200–1000
KHCO_3_	1000
KH_2_PO_4_	20–1300
CaCl_2_·2H_2_O	100
MgSO_4_·7H_2_O	200
Na_2_S_2_O_3_	24.81
Trace element solution 1 (g L^−1^): FeSO_4_·7H_2_O 10, C_10_H_14_N_2_Na_2_O_3_ 5.6	1 mL L^−1^
Trace element solution 2 (g L^−1^): MnCl_2_·4H_2_O 0.352, CoCl_2_·6H_2_O 0.096, NiCl_2_·6H_2_O 0.08, CuSO_4_·5H_2_O 0.1, ZnSO_4_·7H_2_O 0.172, NaSeO_4_·10H_2_O 0.105, NaMoO_4_·2H_2_O 0.11, C_10_H_14_N_2_Na_2_O_3_ 5.0	1 mL L^−1^

### Analytical methods

NO_2_-N and NH_4_-N were measured by the colorimetric method according to Standard Methods (APHA 1995). Total nitrogen (TN) was determined by the persulfate method using the UV spectrophotometric screening method (APHA 1995) for quantification of TN as NO_3_-N (the oxidization product of the persulfate digestion). NO_3_-N (of the original sample) was determined by calculation of the difference of TN and the sum of NO_2_-N and NH_4_-N. The pH was measured by using a pH meter (9010, Jenco, USA), and dissolved oxygen (DO) was measured by using a DO meter (6010, Jenco, USA).

### Gas collection and analysis

Gas was collected through the GSS (Fig. 1) and the volume was measured using an inverted cylinder containing tap water with the pH lowered to 3 using 1-N H_2_SO4. Gas analyses were performed by using a GC-112A gas chromatograph (INESA INSTRUMENT, China).

### DNA extraction and high-throughput 16 s rRNA gene pyrosequencing

After 139 days of operation, the particle based granules were taken out from the Anammox reactor. A granular sludge sample was first ground with a pestle under liquid nitrogen. Meta-genomic DNA was extracted using the E.Z.N.A. Soil DNA Kit (OMEGA Biotec. D5625-01, USA) according to the manufacturer’s instructions. Amplification of the 16S rRNA gene was performed using primers 27F (forward primer: 5′-AGAGTTTGATCCTGGCTCAG-3′) and 533R (reverse primer: 5′-TTACCGCGGCTGCTGGCAC-3′). PCR was carried out according to the following thermocycling parameters: 120 s initial denaturation at 95 °C, 25 cycles of 30 s at 95 °C, 30 s at 55 °C, 30 s at 72 °C, 5 min final elongation at 72 °C, 10 °C until halted by user. Duplicate PCR products were pooled and purified using the AXYGEN gel extraction kit (Axygen, USA) (Feng et al. 2012).

Pyrosequencing was carried out by a 454 Life Sciences Genome Sequencer FLX Titanium instrument (Roche). Sequences were clustered into operational taxonomic units (OTUs) by setting a 0.03 distance limit (equivalent to 97 % similarity) using the MOTHUR program.

## References

[CR1] APHA (1995). Standard method for the examination of water and wastewater.

[CR2] Feng Q, Wang Y, Wang T, Zheng H, Chu L, Zhang C, Chen H, Kong X, Xing X (2012). Effects of packing rates of cubic-shaped polyurethane foam carriers on the microbial community and the removal of organics and nitrogen in moving bed biofilm reactors. Bioresour Technol.

[CR3] Jetten MSM, Cirpus I, Kartal B, van Niftrik LAMP, van de Pas-Schoonen KT, Sliekers O, Haaijer S, van der Star W, Schmid M, van de Vossenberg J, Schmidt I, Harhangi H, van Loosdrecht M, Gijs Kuenen J, Op den Camp H, Strous M (2005). 1994–2004: 10 years of research on the anaerobic oxidation of ammonium. Biochem Soc Trans.

[CR5] Kampschreur MJ, Temmink H, Kleerebezem R, Jetten MS, van Loosdrecht M (2009). Nitrous oxide emission during wastewater treatment. Water Res.

[CR6] Kampschreur MJ, Poldermans R, Kleerebezem R, van der Star WRL, Haarhuis R, Abma WR, Jetten MSM, van Loosdrecht MCM (2009). Emission of nitrous oxide and nitric oxide from a full-scale single-stage nitritation–anammox reactor. Water Sci Technol.

[CR8] Kartal B, Kuenen JG, Van Loosdrecht MCM (2010). Sewage treatment with anammox. Science.

[CR9] Kuenen JG (2008). Anammox bacteria: from discovery to application. Nat Rev Microbiol.

[CR10] Longfei R, Shuang L, Huu HN, Wenshan G, Shouqing N, Cui L, Yuankun Z, Daisuke H (2015). Enhancement of anammox performance in a novel non-woven fabric membrane bioreactor (nMBR). RSC Adv.

[CR11] Mulder A, Van de Graaf AA, Robertson LA, Kuenen JG (1995). Anaerobic ammonium oxidation discovered in a denitrifying fluidized bed reactor. FEMS Microbiol Ecol.

[CR12] Okabe S, Oshiki M, Takahashi Y, Satoh H (2011). N_2_O emission from a partial nitrification–anammox process and identification of a key biological process of N_2_O emission from anammox granules. Water Res.

[CR13] Shaw AR, Koh SH (2012). Gaseous emissions from wastewater facilities. Water Environ Res.

[CR14] Strous M, Heijnen JJ, Kuenen JG, Jetten MSM (1998). The sequencing batch reactor as a powerful tool for the study of slowly growing anaerobic ammonium-oxidizing microorganisms. Microbiol Biotechnol.

[CR15] Wenjie Z, Yuanyuan Z, Liang L, Xuehong Z, Yue J (2014). Fast start-up of expanded granular sludge bed (EGSB) reactor using stored Anammox sludge. Water Sci Technol.

[CR16] Wenjie Z, Huaqin W, Joseph DR, Yue J (2015). Granular activated carbon as nucleus for formation of Anammox granules in an expanded granular-sludge-bed reactor. Global NEST J.

[CR17] Wunderlin P, Siegrist H, Joss A (2013). Online N_2_O measurement: the next standard for controlling biological ammonia oxidation?. Environ Sci Technol.

[CR18] Yue J, Dunqiu W, Wenjie Z (2015). Use of bamboo charcoal reduced the cultivated anammox seed sludge dosage during the start-up period. Desalin Water Treat.

